# 3D printed versus milled stabilization splints for the management of bruxism and temporomandibular disorders: study protocol for a randomized prospective single-blinded crossover trial

**DOI:** 10.1186/s13063-024-08437-7

**Published:** 2024-09-05

**Authors:** Kerstin Rabel, Jörg Lüchtenborg, Marie Linke, Felix Burkhardt, Anuschka J. Roesner, Julian Nold, Kirstin Vach, Siegbert Witkowski, Anna-Lena Hillebrecht, Benedikt C. Spies

**Affiliations:** 1 Department of Prosthetic Dentistry, Center for Dental Medicine, Medical Center ‑ University of Freiburg, Faculty of Medicine, University of Freiburg, Hugstetterstr. 55, 79106 Freiburg, Germany; 2 Institute of Medical Biometry and Statistics, Medical Center ‑ University of Freiburg, Faculty of Medicine, University of Freiburg, Stefan-Meier-Str. 26, Freiburg, 79104 Germany

**Keywords:** Digital dentistry, Stabilization splint, CAD/CAM, Additive manufacturing, 3D printing, Milling, Bruxism, Temporomandibular disorder, Oral health-related quality of life

## Abstract

**Background:**

Nowadays, stabilization splints for the management of bruxism and temporomandibular disorders (TMD) can be produced utilizing a digital workflow comprising a digital impression of the teeth, digital splint design, and computer-aided manufacturing of the splints. The latter is usually a milling process, however, more recently 3D printing gained popularity due to its better cost and time efficiency. It remains unknown whether 3D printed stabilization splints are inferior to milled splints regarding clinical outcomes.

**Methods:**

This clinical trial assesses the non-inferiority of 3D printed occlusal splints compared to milled occlusal splints in a monocentric prospective randomized single-blinded crossover trial with two cohorts. One cohort includes 20 participants with bruxism, the other 20 participants with pain-related TMD, i.e., myalgia, myofascial pain, or arthralgia of the jaw muscles/the temporomandibular joint(s) diagnosed according to the Diagnostic Criteria for Temporomandibular Disorders (DC/TMD). Michigan-type stabilization splints are fabricated in a digital workflow by milling or 3D printing using CE-marked materials within their intended purpose. The participants wear a milled and a 3D printed splint in a randomized order for 3 months each, with follow-up visits after 2 weeks and 3 months. Investigated outcome parameters are oral health-related quality of life (OHRQoL) evaluated by the Oral Health Impact Profile (OHIP-G14), participant satisfaction as rated on a visual analog scale, therapeutic efficacy, and technical result of the splints. In this context, therapeutic efficacy means antagonist wear and—in the TMD group—reduction of pain/disability assessed by the Graded Chronic Pain Scale (GCPS v2.0) and clinical assessment following the DC/TMD standard, while technical outcome measures splint fit, wear and fracture rate.

**Discussion:**

The trial will provide important information on the clinical outcome of 3D printed stabilization splints in comparison to milled splints and will, therefore, enable an evidence-based decision in favor of or against a manufacturing process. This, in turn, will guarantee for a maximum of the patient’s OHRQoL during splint therapy, therapeutic efficacy, and longevity of the splints.

**Trial registration:**

German Clinical Trials Register (DRKS) DRKS00033904. Registered on March 15, 2024.

## Administrative information

**Table:** 

Title {1}	3D printed versus milled stabilization splints for the management of bruxism and temporomandibular disorders: study protocol for a randomized prospective single-blinded crossover trial
Trial registration {2a and 2b}	The trial is registered in the German Clinical Trials Register (DRKS) with the registration number DRKS00033904.
Protocol version {3}	Protocol version 1.1 from the 22^nd^ of December 2023
Funding {4}	This work was funded by the Berta-Ottenstein-Program for Advanced Clinician Scientists, Faculty of Medicine, University of Freiburg.
Author details {5a}	Kerstin Rabel^1^ Jörg Lüchtenborg^1^ Marie Linke^1^ Felix Burkhardt^1^ Anuschka J. Roesner^1^ Julian Nold^1^ Kirstin Vach^2^ Siegbert Witkowski^1^ Anna-Lena Hillebrecht^1^ Benedikt C. Spies^1^ 1 Department of Prosthetic Dentistry, Center for Dental Medicine, Medical Center ‑ University of Freiburg, Faculty of Medicine, University of Freiburg, Hugstetterstr. 55, 79106 Freiburg, Germany 2 Institute of Medical Biometry and Statistics, Medical Center - University of Freiburg, Faculty of Medicine, University of Freiburg, Stefan-Meier-Str. 26, 79104 Freiburg, Germany
Name and contact information for the trial sponsor {5b}	The present trial is an Investigator Initiated Trial, for contact information see author KR.
Role of sponsor {5c}	Since it is an Investigator Initiated Trial, the trial was designed, is conducted and published by the authors listed above.

## Introduction

### Background and rationale {6a}

Bruxism and temporomandibular disorders (TMD) are common complaints for which patients consult their dentist, with prevalence rates in the adult population exceeding 30% and 5%, respectively [1–3]. The term bruxism refers to an “oromotor behavior” defined as “repetitive jaw-muscle activity characterized by clenching or grinding of the teeth and/or by bracing or thrusting of the mandible.” [4–8]. Bruxism becomes clinically relevant when it leads to destructive teeth wear, failure of dental restorations, teeth, and/or implants and when hypertrophied masticatory muscles impair facial esthetics [6, 9]. Further, bruxism was described as one factor that can contribute to TMD genesis [6]. TMD represents a heterogeneous group of disorders involving the temporomandibular joints (TMJ) and/or jaw muscles with symptoms including TMJ sounds, TMJ and/or jaw muscle pain, and restriction, deviation, or deflection of the mouth opening path [10, 11]. After chronic low back pain, TMD are the second most common musculoskeletal disorder causing pain and disability, with an annual cost of $4 billion in the US [12].

For the management of bruxism and pain-related TMD—referring to myalgia, myofascial pain or arthralgia of the jaw muscles and TMJ(s)—stabilization splints, also known as Michigan-type splints, are employed [6, 13]. These splints are characterized by the following features: they are made from hard acrylic resin, they cover all teeth in the splint-bearing jaw, the posterior teeth of the opposing jaw have even and simultaneous contact with the splint with a flat surface and cuspid rise during splint-guided laterotrusion and protrusion starts after up to 1 mm freedom in centric [14–16]. The therapeutic rationale for stabilization splint usage in individuals with bruxism is the prevention or limitation of dental damage possibly caused by the disorder [6]. Wearing a stabilization splint further results in a change of the functional muscular patterns due to an increase in vertical distance between the upper and lower jaw leading to altered load distribution in the TMJ and jaw muscles [13, 15, 17]. This relieves overstressed areas of these structures and alleviates pain [13, 15, 17].

Nowadays, stabilization splints can be fabricated in a digital workflow, during which they are virtually designed based on a digital impression of the jaws before they are fabricated using computer-aided manufacturing (CAM). The digital workflow is more time efficient and leads to a better fit and patient comfort than the conventional workflow comprising alginate/silicone impressions, cast fabrication, wax-up, and molding the splint from powder-liquid mixtures of polymethylmethacrylate (PMMA) [18–20]. CAM is usually a subtractive procedure during which the splint is milled from an industrially manufactured PMMA blank. From these blanks, 25–30 wt.% are milled away during splint fabrication, 70–75 wt.% remain unused around the splint and are subsequently discarded while the splint itself only accounts for 2–5 wt.% of the blank. Additionally, burs used for milling require frequent exchange since they deteriorate rapidly. Milling, therefore, requires an enormous amount of material and is hence neither cost nor material efficient and has a negative impact on the environment. In recent years, 3D printing has become increasingly popular for splint fabrication. The term “3D printing” is the most commonly used umbrella term in biomedical research for processes in which objects are built additively layer by layer based on a digital file [21, 22]. 3D printing enables the production of more complex geometries with better resource, cost, and time efficiency in comparison to the established milling process. The reason for this is that the ratio of the feedstock material to the material of the final object is higher compared to milling. Furthermore, a parallel production of objects is possible [23–25]. 3D printed stabilization splints may therefore reduce the economic burden and ecological implications of bruxism and TMD management with milled splints. However, the precondition for the production-specific advantages of 3D printing to unfold is that 3D printed splints are not clinically inferior to milled splints. Key clinical outcome parameters for a successful splint therapy are (i) patients' oral health-related quality of life (OHRQoL) with the splints, (ii) therapeutic efficacy, i.e., abrasion of the opposing dentition and pain reduction, and (iii) technical outcome, i.e., fit, wear and fracture rate of 3D printed splints. Due to the unavailability of data on these parameters for 3D printed splints, it is at present not possible to assess a potential inferiority of 3D printed stabilization splints compared to milled stabilization splints.

### Objectives {7}

Therefore, the objective of this clinical trial is to analyze whether the clinical outcome of 3D printed stabilization splints is inferior compared to milled stabilization splints in terms of patients´ OHRQoL and satisfaction with the splints, therapeutic efficacy, and technical outcome. These parameters will be investigated in both—a cohort comprising participants with bruxism and a cohort with participants suffering from pain-related TMD—over a period of 6 months. The null hypothesis states that novel 3D printed stabilization splints for use in bruxism or TMJ and/or jaw muscle pain are not inferior to milled stabilization splints with regard to the aforementioned parameters.

### Trial design {8}

The clinical trial to assess the non-inferiority of 3D printed stabilization splints compared to milled stabilization splints is designed as a monocentric prospective randomized single-blinded crossover trial with two cohorts. According to the indication spectrum of stabilization splints, one cohort comprises 20 participants with bruxism whereas the other arm is formed by 20 participants with pain-related TMD. Only CE-labeled materials are used within their intended purpose for the fabrication of milled and 3D printed splints. In both cohorts, the treatment sequence, meaning that participants either receive a milled splint first and then a 3D printed splint or vice versa is randomized and allocated at a ratio of 1:1. The 3D printed and milled stabilization splints are each worn for 3 months in an order that is blinded to the participants.

## Methods: participants, interventions, and outcomes

### Study setting {9}

The clinical trial is performed at the Department of Prosthetic Dentistry, Center for Dental Medicine, Medical Center, University of Freiburg, Germany.

### Eligibility criteria {10}

For both cohorts, only patients who are being treated at the above-mentioned Department of Prosthetic Dentistry, who are older than 18 years, and who do not require special protection for other reasons are eligible. Treatment with a Michigan-type stabilization splint for the upper jaw to be worn at night is indicated to relieve symptoms of bruxism or TMD. The patient’s state of health must permit dental treatment and patients must have good knowledge of spoken and written German. Patients are only included in the trial if they give written informed consent. General exclusion criteria are known allergies to materials used in the study and drug or alcohol abuse.

Further inclusion and exclusion criteria are specified for the two cohorts as follows:

- Bruxism cohort:
  - Inclusion criteria: The bruxism screening index (BSI) of the German Society of Craniomandibular Function and Disorders (DGFDT) must hint at a probable bruxism.
  - Exclusion criteria: Patients presenting with chief compliant orofacial pain and diagnosed with myalgia or myofascial pain of the jaw muscles and/or arthralgia of the TMJ(s) according to the Diagnostic Criteria for Temporomandibular Disorders (DC/TMD) are excluded from this cohort. Additionally, patients are excluded if they underwent therapies for bruxism that could interfere with the splint treatment, including the insertion of a splint in the month preceding the trial or injections of botulinum toxin into the jaw muscles in the 6 months preceding the trial.
- TMD cohort:
  - Inclusion criteria: The patients seek medical treatment because of pain located in the TMJ and/or jaw muscles. Patients are included in the clinical trial if a myalgia or myofascial pain of the jaw muscles and/or arthralgia of the temporomandibular joint(s) according to the DC/TMD are diagnosed and if the Graded Chronic Pain Scale version 2.0 (GCPS) adopts a maximum of grade II.
  - Exclusion criteria: Patients with GCPS grade III or IV are excluded from the clinical trial. Furthermore, patients with orofacial pain of dental, traumatic, or systemic origin requiring medical treatment other than or prior to splint therapy are excluded. In addition, patients are excluded if they received treatment for TMD that could potentially affect the outcome of the splint treatment, such as insertion of a splint in the month prior to the trial, or injections of botulinum toxin into the jaw muscles in the 6 months prior to the trial. TMJ(s) should not show any pain on traction.

The patients of the TMD cohort are diagnosed and treated by dentists who have more than 7 years of clinical experience in the field of prosthetic dentistry and TMD and who have attended structured postgraduate programs in TMD. The dentists were trained and calibrated in the clinical application of the DC/TMD by gold standard examiners in a course of the INfORM Training and Reliability center, University of Leipzig, Germany. The bruxism cohort is treated by a doctorate candidate in dentistry who is supervised by a dentist from the TMD cohort.

### Who will take informed consent? {26a}

When patients matching the eligibility criteria are interested in participating in the clinical trial, they are informed by study personnel in detail about the background, aims, treatment protocol, data collection and processing as well as risks and benefits of the trial with the help of a patient information leaflet approved by the Ethics Committee of the Albert-Ludwigs-University, Freiburg, Germany. The patients are encouraged to ask any further questions during this interview. If patients do not have any further questions and wish to participate in the trial, an informed consent form is signed by the participant and the person conducting the informed consent interview prior to digital impression taking.

### Additional consent provisions for collection and use of participant data and biological specimens {26b}

N/a since no biological specimens are collected and since no use of participant data in ancillary studies is planned.

## Interventions

### Explanation for the choice of comparators {6b}

Milled splints are used as comparators as subtractive manufacturing represents the standard CAM fabrication technique for splints. Another option would have been to use splints fabricated conventionally by alginate/silicone impression, cast fabrication, wax-up, and molding from powder-liquid mixtures of PMMA as comparators. It was decided to use milled splints as a comparator since the digital procedure will replace conventional processes due to better time-efficiency and patient comfort [18–20]. In addition, the comparison of two splints fabricated using different CAM processes enables the comparison of splints based on the same digital design and therefore allows differences in splint design/shape to be ruled out as the cause of possible differences in clinical outcome. In contrast, conventionally fabricated and digitally fabricated splints would differ in splint design/shape rendering direct comparison of the effects of the fabrication mode on clinical outcome infeasible.

### Intervention description {11a}

Participants are examined and diagnosed following standard protocols of the DGFDT and the DC/TMD (T0). The BSI, symptom questionnaire of the DC/TMD, the DC/TMD examination form, and the GCPS are used for this purpose. Afterwards, digital impressions of the participants’ upper and lower jaw are taken with an intraoral scanner (Trios 4, 3Shape, Kopenhagen, Denmark), and therapeutic relation, often entitled “centric” relation, is registered as described by Türp [26] with wax sheets at the intended height of the splint (in most cases approx. 2 mm interocclusal space in molar regions). Lateral bite scans are then performed with the intraoral scanner and the adapted wax sheet in situ. Based on this data set, the Michigan-type stabilization splints are designed with the software SplintStudio (3Shape). Then, based on this design, one occlusal splint is 3D printed from Primeprint splint (Dentsply Sirona, Bensheim, Germany; the material is marketed by Dentsply Sirona under the name Primeprint Splint, but is manufactured by DETAX, Ettlingen, Germany as Freeprint Splint 2.0, i.e., Primeprint splint and Freeprint Splint 2.0 are the same materials and both have the UDI DI EDET0465W). The 3D printing process is carried out using a Primeprint printer (Dentsply Sirona). Subtractively manufactured splints are milled from Ceramill A-Splint blanks (Amann Girrbach, Pforzheim, Germany) with an inLab MC X5 (Dentsply Sirona, Bensheim, Germany). The materials are processed as specified by the manufacturers. Participants are instructed to wear the splints at night.

Starting with the incorporation of the first splint, the trial is carried out according to the following protocol:

- Incorporation of the 1^st^ splint (T1): scan of the splint as delivered from the laboratory with the intraoral scanner and—if necessary—also after adjustment, completion of the Oral Health Impact Profile (OHIP-G14, German version from 2005) by all participants of both cohorts, completion of the GCPS by participants of the TMD cohort, examination of the TMD patients according to the DC/TMD standard
- Control 2 weeks after incorporation of 1^st^ splint (T2): if necessary, adjustment and scan of the splint, completion of the OHIP-G14 and GCPS and examination of TMD patients as described above, rating of wear comfort and retention of the stabilization splints on a VAS scale of 1–10 by participants of both cohorts
- Control 3 months (11–13 weeks) after incorporation of the 1^st^ splint (T3): scan of the splint and opposing dentition with the intraoral scanner, completion of the OHIP-G14 and GCPS, examination of TMD patients and rating of wear comfort and retention as described for T2, scan of the 2^nd^ splint as delivered from the laboratory and—if necessary—also after adjustment, incorporation of the 2^nd^ splint.
- Control 2 weeks after incorporation of 2^nd^ splint (T4): see T2
- Control 3 months (11–13 weeks) after incorporation of the 2^nd^ splint (T5): see T3. Additionally, the participant is asked to name his or her favorite splint (1^st^ or 2^nd^)

A wash-out period between the two splints was not included, as this may lead to aggravation of symptoms and therefore bears the risk that patients may switch to alternative treatment options such as commercially available splints to alleviate symptoms during the wash-out period, rendering trial conditions less controlled.

### Criteria for discontinuing or modifying allocated interventions {11b}

Criteria for discontinuing or modifying allocated interventions for a given trial participant are the occurrence of exclusion criteria for the respective cohort or the withdrawal of consent. Interventions may also be discontinued if a participant unexpectedly experiences a worsening of symptoms that the participant considers intolerable.

### Strategies to improve adherence to interventions {11c}

From the strategies to improve adherence in clinical research proposed by Robiner [27] and Matsui [28], the following aspects are considered in this trial: the study personnel spends sufficient time during the first examination to assess the participants’ potential adherence. The protocol, the benefits for (future) patients, and the importance of adherence are explained in detail to participants to help them understand the objectives of the trial and the importance of their adherence. Participants are explicitly informed about when and how to wear the splint. The appointments are scheduled at convenient times and the splints are offered free of charge to compensate for the time required to participate in the trial. Adherence is monitored by self-report of the participants. In general, participants can be assumed as intrinsically motivated to wear the splints, since this will reduce teeth abrasion and/or pain.

### Relevant concomitant care permitted or prohibited during the trial {11d}

During participation in the trial, participants should not undergo concomitant treatments or interventions like botulinum toxin injections into masticatory muscles, pain therapy with prescription painkillers, muscle relaxants, or physiotherapy. If any of these measures are necessary for medical reasons, further participation in the trial will be discussed on an individual basis. Short-term use of over-the-counter painkillers is permitted as well as application of heat/cold, self-massage, and continuation of physiotherapy exercises already learnt prior to the trial.

### Provisions for post-trial care {30}

Only established treatment modalities with a very low risk for adverse events are applied and participants can remove the splints themselves when they feel uncomfortable with them. Harm induced by trial participation, therefore, appears improbable which is the reason why no compensations for harm suffered from trial participation are deemed necessary. Participants can keep both splints after trial participation and are invited to regular controls at our department.

### Outcomes {12}

#### Primary outcomes

As the primary outcome, the OHRQoL of the participants is assessed using the OHIP-G14 questionnaire on the day of incorporation and after 2 weeks and 3 months after incorporation of each splint. Depending on the answer options ticked by the participants, this questionnaire results in scores between 0 and 56. For each participant and splint, the difference from the baseline (incorporation of splint) is evaluated at 2 weeks and 3 months post insertion.

Additionally, participant satisfaction with the stabilization splints is investigated by asking the questions “How comfortable was wearing the splint for you on a scale from 1 (very uncomfortable) to 10 (very comfortable)?” and “How stable did the splint rest in your mouth at a scale from 1 (very unstable) to 10 (very stable)?”. These questions are asked at the 2 weeks and 3 months controls and are analyzed for differences in relation to the manufacturing method.

#### Secondary outcomes

Secondary outcomes are treatment efficacy and technical results of the stabilization splints. In the present trial, treatment efficacy is defined as the extent of abrasion of the opposing dentition in the bruxism cohort since the reduction of teeth abrasion is the therapeutic rationale for splint incorporation in bruxers. Teeth abrasion is measured as volume loss of the opposing dentition by superimposing intraoral scans obtained at digital impression taking and 3 months control appointments. Treatment efficacy in the TMD cohort is additionally investigated by quantification of pain reduction as the lead symptom of pain-related TMD. For this, the GCPS is evaluated on the day of incorporation and control appointments. The differences in the values obtained for chronic pain intensity (point score between 0 and 100), disability days (between 0 and 30), and interference score (point score 0–100) at control appointments and incorporation days are then calculated for every participant and splint type. Further, the number of painful points on examination according to the DC/TMD is compared at control appointments versus the incorporation date (range 0 to 48). Jaw mobility is investigated by measuring maximum jaw opening, laterotrusion, and protrusion in millimeters at incorporation and control appointments.

In addition, the technical results of 3D printed and milled stabilization splints are compared. Fit at incorporation is evaluated by calculating the amount of adjustment necessary in µm^3^ by overlaying scans before and after adjustment of the splint. Wear of the occlusal splints is assessed by calculating volume loss in µm^3^ by overlaying scans of the splints obtained at incorporation and the 3 months control. The integrity of the occlusal splints (no fracture of the occlusal splint) is assessed at control appointments, and the rate of fractures occurring is calculated in percent for each type of splint.

### Participant timeline {13}

**Fig. 1:**
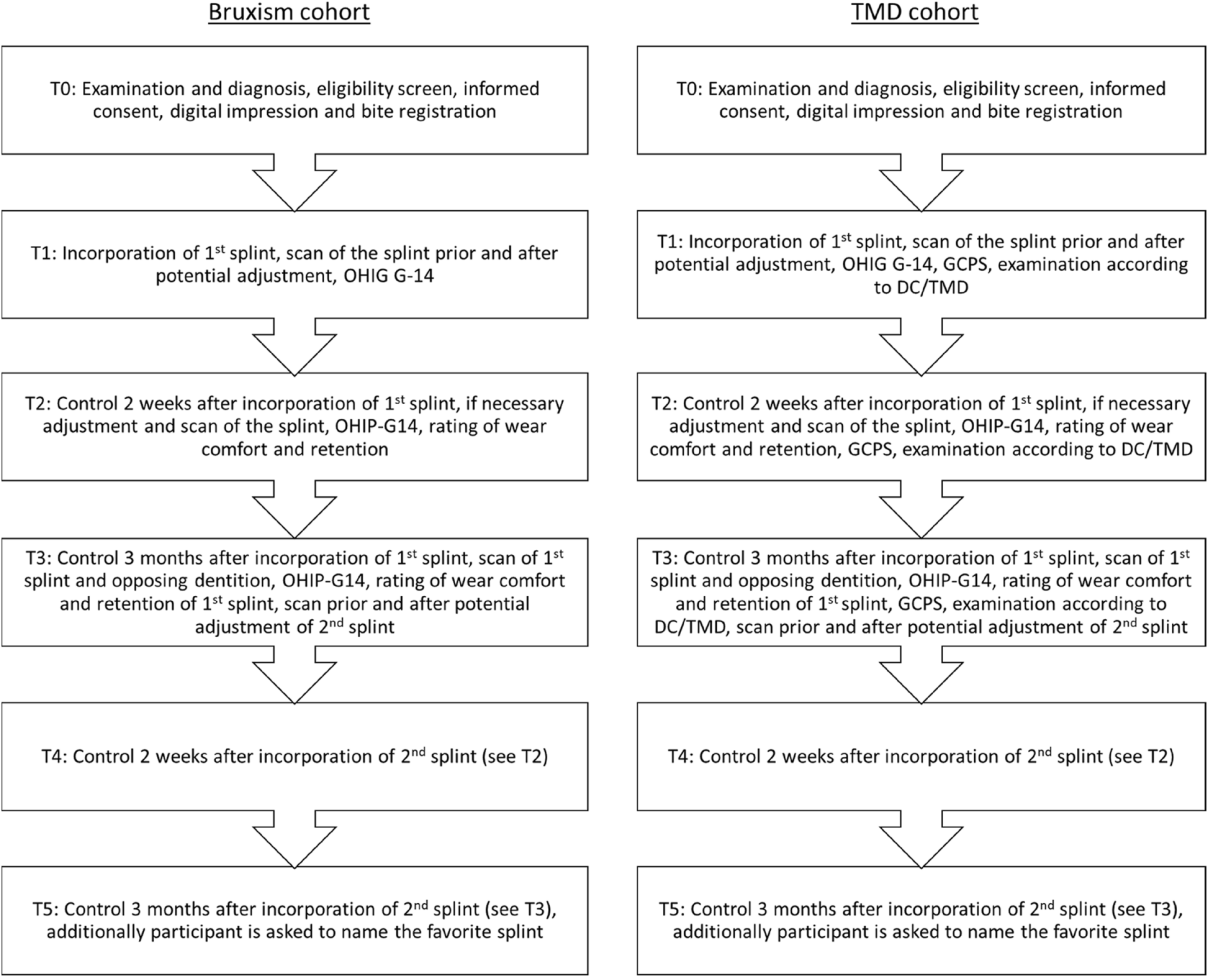
Participant timeline. Sequence of splints (3D printed or milled) is randomized at an allocation ratio of 1:1

**Fig. 2:**
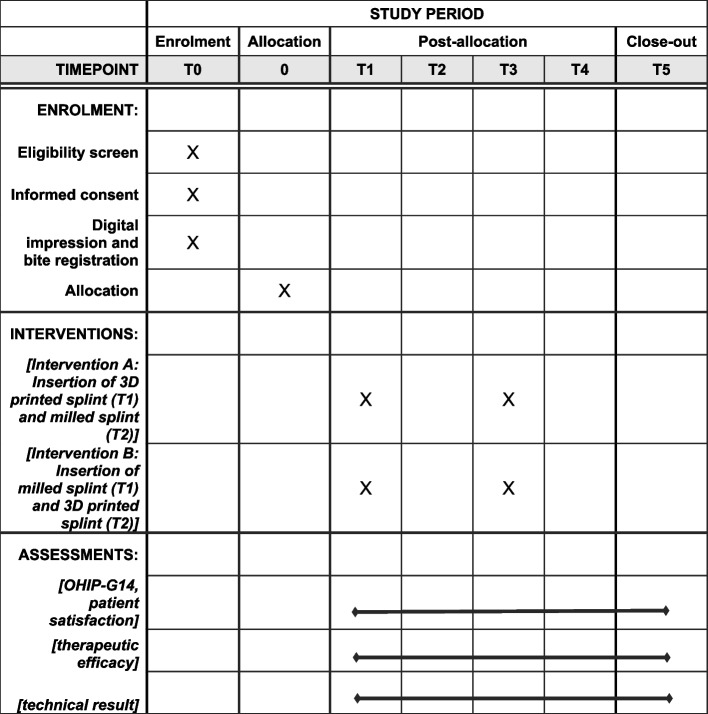
Standard protocol items diagram according to the SPIRIT guidelines

The participant timeline is depicted in Fig. 1. Additionally, Fig. 2 illustrates the standard protocol items diagram according to the guidelines proposed by Standard Protocol Items: Recommendations for Interventional Trials (SPIRIT).

### Sample size {14}

The sample size was calculated based on results reported for the OHIP-G14. According to the results of Alajbeg et al. [29], an OHIP-G14 score of 25.29 (SD 12.38) can be assumed for TMD patients. If the non-inferiority margin is set at 5 and a standard deviation of the differences of 10 is assumed, 38 participants are required for the trial with a two-sided paired t-test and a power of 90% on a 5% significance level. As both cohorts can be analyzed together for the primary outcome parameter OHIP-G14, the target number of cases is set at 20 participants per cohort, resulting in 40 participants in total.

### Recruitment {15}

Dentists of the Department of Prosthetic Dentistry, Medical Centre, University of Freiburg, Germany screen patients for trial eligibility. No further strategies for achieving adequate participant enrolment are conducted to reach the target sample size since both—bruxism and TMD—are frequent in the patient population of the above-mentioned clinic.

## Assignment of interventions: allocation

### Sequence generation {16a}

The order of the splints, which can be either a milled splint first and then a 3D printed splint or vice versa, is assigned by simple randomization in a 1:1 ratio according to a computer-generated randomization scheme. This randomization scheme is created by the GraphPad Web calculator to randomly assign subjects to treatment groups.

### Concealment mechanism {16b}

Treatment order is concealed for trial participants but not for study personnel involved in treating participants since 3D printed and milled splints can be distinguished by trained clinicians.

### Implementation {16c}

The allocation scheme was generated at the start of the trial by the principal investigator (PI) who also enrolls participants. Since all participants receive the same intervention, the explanation of who will assign participants to interventions is n/a.

## Assignment of interventions: blinding

### Who will be blinded {17a}

Participants are blinded since they are not told how the splint was fabricated. Further, the statistician analyses the data generated during the trial blinded. Treating dentists cannot be blinded since trained personnel can differentiate between a 3D printed and milled splint.

### Procedure for unblinding if needed {17b}

If deemed necessary, unblinding may be performed by the study personnel who can visually differentiate between the milled and 3D printed splints. Unblinding of the participant will only be performed if it is essential for further management of the participant, for example in the case of severe allergic reactions requiring further assessment of the allergy-initiating factors.

## Data collection and management

### Plans for assessment and collection of outcomes {18a}

Data of the TMD cohort is collected and assessed by one trained and calibrated examiner according to the DC/TMD protocol. This examiner also trained the examiner collecting and assessing data of the bruxism cohort. Data will be collected via established diagnosis tools and patient questionnaires (BSI, DC/TMD symptom questionnaire, DC/TMD examination form, GCPS, OHIP-G14). These forms can be found on the following webpages:

- BSI can be downloaded from the webpage of the DGFDT (https://www.dgfdt.de/richtlinien_formulare)
- DC/TMD symptom questionnaire, DC/TMD examination form and GCPS (version 2.0) can be found in different languages on the webpage of the International Network for Orofacial Pain and Related Disorder Methodology (INfORM—https://ubwp.buffalo.edu/rdc-tmdinternational/tmd-assessmentdiagnosis/dc-tmd-translations/). Specifications on how to assess and diagnose a patient in a standardized way can also be found on this webpage. This trial applied the German forms.
- OHIP-G14 can be accessed in German via the Institute of German Dentists (IDZ- https://www.idz.institute/fileadmin/Content/Publikationen-PDF/IDZ-2005-Fragebogen_OHIP-G-14.pdf).

The completed questionnaires are then assessed as specified by the DGFDT and INfORM. All forms are standard forms and are commonly applied in the context of bruxism and TMD. Validity and reliability of the DC/TMD are well characterized. A myalgia, myofascial pain, and arthralgia can be diagnosed with a sensitivity of ≥ 0.86 and a specificity of ≥ 0.98 and with an inter-examiner reliability for the clinical assessment of kappa ≥ 0.85 [30]. The GCPS is recognized as a sensitive tool to detect minor changes in TMD symptoms [20] and OHIP-G14 shows a correlation with self-perception of oral health and physical and mental dimensions and shows satisfactory validity and reliability [31, 32].

Furthermore, the volume change of splints and opposing dentition is calculated by overlaying optical scans with the software Geomagic Control X (3D Systems, Moerfelden-Walldorf, Germany) by trained users of the software.

### Plans to promote participant retention and complete follow-up {18b}

Participants are informed about the importance of their adherence and are motivated to follow trial guidelines. Further, participants receive the two splints for free to promote participant retention and complete follow-up. If a participant drops out of the trial or deviates from intervention protocols, the reason for discontinuation and deviation from the protocol will be recorded and it will be assessed on an individual basis if and how data generated during the trial can be implemented in statistical analysis.

### Data management {19}

Data is managed according to established protocols at the Medical Center, University of Freiburg meaning that treatment-relevant data is entered and stored electronically and revision safe in software that can only be accessed by personnel of the clinic via password from inside clinic buildings and via two-factor authentication from remote workstations. Data collected only for trial purposes is stored separately and can only be accessed by study personnel. This trial data is stored pseudonymized, meaning that the participant identifiers are replaced by a number, at the server of the aforementioned clinic. The list providing information on the allocation of numbers and participant identifiers is stored separately. If data needs to be transferred from one computer to another or from the intraoral scanner to a computer, the cloud provided by above-mentioned clinic is used for data transfer. Alternatively, if data transfer via cloud is not possible, encrypted mobile data storage media are used.

### Confidentiality {27}

All data of the participants is treated confidentially by the trial’s personnel. Data is collected via digital questionnaires and established assessment forms or is digitized after filling in paper documents. Then, data is stored as described above.

### Plans for collection, laboratory evaluation, and storage of biological specimens for genetic or molecular analysis in this trial/future use {33}

N/a because no biological specimens are collected.

## Statistical methods

### Statistical methods for primary and secondary outcomes {20a}

Absolute and relative frequencies are calculated for binary or categorical variables. For continuous data, the median, mean and standard deviation are specified. In addition, 95% confidence intervals are calculated. The calculations are performed per group (milled and 3D printed) and per cohort (bruxism and TMD). Cross-over effects are examined in all analyses. For the primary outcome parameter OHIP-G14, the group differences are estimated and tested using a linear regression model with corresponding two-sided 95% confidence intervals. The model will include the difference in OHIP-G14 values (milled vs. 3D printed) as a dependent variable and the sequence of the splints and the cohort (bruxism and TMD) as independent variables.

The one-sided test for non-inferiority of 3D printed versus milled splints at the 2.5% significance level is based on the two-sided 95% confidence interval of the linear regression model. The null hypothesis is rejected if the lower limit of the confidence interval for the difference (conventionally milled vs. 3D printed) is above − 5. The same applies to secondary outcomes, namely volume loss of the splint/opposing dentition, pain reduction, jaw mobility, and splint fracture rates.

### Interim analyses {21b}

N/a no interim analyses are planned due to the minimal risks of the trial.

### Methods for additional analyses (e.g., subgroup analyses) {20b}

N/a. No additional analyses are currently planned.

### Methods in analysis to handle protocol non-adherence and any statistical methods to handle missing data {20c}

Reasons for trial discontinuation will be noted for each randomization group and the reasons will be analyzed for dependence from the randomization group and fabrication mode of the splint. Imputation of missing data is not planned; if unexpectedly a larger number of participants drops out during the trial, further participants will be enrolled to obtain the intended number of participants required for a statistical power of 90%.

### Plans to give access to the full protocol, participant-level data, and statistical code {31c}

The full protocol approved by the Ethics Committee (in German language) and the anonymized dataset for statistical analysis as well as information concerning the statistical analysis can be obtained from the corresponding author on reasonable request.

## Oversight and monitoring

### Composition of the coordinating center and trial steering committee {5d}

N/a since all work related to the trial—enrolling and treating participants, fabrication of splints, and data analysis—is performed at the Department of Prosthetic Dentistry, Center for Dental Medicine, Medical Center, University of Freiburg, Germany. A coordinating center and trial steering committee were not planned due to the monocentric character of the trial and its low risks for the participants.

### Composition of the data monitoring committee, its role and reporting structure {21a}

N/a because a data monitoring committee was deemed not necessary since this clinical trial involves only the use of established and approved materials within their intended purpose, therefore, risks for participants can be regarded as minimal.

### Adverse event reporting and harms {22}

In the time between the incorporation of the first splint and the 6 months control, adverse events and serious adverse events as defined in the Medical Device Regulation (MDR) Article 2, points 57 and 58 are recorded by the trial´s personnel and are assessed for a causal relationship with the applied milled and 3D printed materials. Further, device deficiency (MDR, Article 2, point 59) will be noted in the aforementioned time frame. However, since no additional invasive and/or burdensome procedures are included in the trial, the trial represents an “other clinical investigation” according to German law (Section 64(4) Medizinproduktedurchführungsgesetz (MDPG)). Therefore, (serious) adverse events and device deficiency do not have to be reported to the federal higher authority.

The Ethics Committee of the Albert-Ludwigs-University, Freiburg, Germany will be informed about potential serious adverse events with assumed causal relationship to the clinical trial. Since the investigational materials have CE marking, are processed as suggested by the manufacturers, and are used within the intended purpose, (serious) adverse events are not expected during the trial.

### Frequency and plans for auditing trial conduct {23}

N/a since no audit from an independent party is planned in this Investigator Initiated Trial. The clinical trial will be closely monitored by the trial’s personnel and persons involved in participant treatment will meet regularly to discuss the trial’s progress and potential issues arising during the trial.

### Plans for communicating important protocol amendments to relevant parties (e.g. trial participants, ethical committees) {25}

Protocol amendments will be submitted for review to the Ethics Committee of the Albert-Ludwigs-University, Freiburg, Germany, and will only be implemented in the trial after approval. Entries in trial registries will be updated accordingly and—if trial participants are affected by the amendment—they are informed personally.

### Dissemination plans {31a}

Data will be disseminated through peer-reviewed scientific journals and at clinical and academic conferences. No publication restrictions exist.

## Discussion

3D printing is becoming increasingly popular in dentistry as more complex geometries can be produced with better cost and time efficiency compared to the established milling procedure. Due to these production-specific advantages of 3D printing, a growing number of stabilization splints is fabricated using 3D printing. However, it remains currently unknown whether these 3D printed stabilization splints perform as successfully clinically as milled splints.

The presented trial, therefore, investigates the non-inferiority of 3D printed stabilization splints compared to milled counterparts in terms of OHRQoL, treatment efficacy, and technical result in a monocentric prospective randomized single-blinded crossover trial with a bruxism and a TMD cohort. It was decided to apply a cross-over design to allow for direct comparison of treatment effects of milled and 3D printed splints on the same individual which can be assumed as more precise than a parallel design in this setting [33]. To reduce the carry-over-effects typical for cross-over designs, two arms with different treatment sequences are investigated per cohort, participants are blinded and cross-over effects will be examined in all analyses [33, 34].

Clinical outcomes of the splints are investigated in a bruxism and TMD cohort, since both conditions can be managed by stabilization splints. Bruxism and TMD show large inter-individual variation due to their multifactorial biopsychosocial genesis [15]. There is thus a continuum spectrum for bruxism ranging from physiologic motor action regarded as self-protective behavior of which patients are often unaware to severely destructive bruxism resulting in serious orofacial health problems and tooth decay [8, 9, 35, 36]. Bruxism can further contribute to the genesis of TMD [35, 36] which itself can show symptoms from pain-free TMJ sound to dysfunctional chronic orofacial pain and disability [30]. As a consequence, the definition of bruxism and TMD for trial purposes, which determines participant inclusion and comparability of clinical trials, is challenging. Definitive diagnosis of bruxism requires an instrumental approach using polysomnography including electromyography, which cannot be used routinely in clinical practice or research due to its technical complexity, high cost, and limited availability [5, 37, 36]. Hence, a probable bruxism, referring to bruxism diagnosed by clinical dental examination [5, 37, 36], was set as the inclusion criterion for this trial. For diagnosis of a probable bruxism, different screening instruments have been proposed but no internationally established screening existed at initiating the present trial. Therefore, the authors apply the BSI representing the standard tool applied in Germany for bruxism screening to identify individuals with probable bruxism [36]. The BSI is in many aspects comparable to the BruxScreen developed for setting an international standard in bruxism screening and initially tested by Lobbezoo et al. [35] when the present trial was planned and might thus yield comparable results. Future research comparing the results of different clinical screenings for probable bruxism and the establishment of an international standard for probable bruxism diagnosis will benefit the comparability of clinical trials in this field.

For the diagnosis of TMD, the DC/TMD, primarily established as RDC/TMD in 1992 and updated in 2014, can be regarded as the standard assessment and diagnostic tool [30]. For this reason, participants are examined and diagnosed following the guidelines of the DC/TMD in the present trial. Only pain-related TMDs are included in the trial as these conditions—but not pain-free intra-articular TMD—can be effectively managed by stabilization splints. These diagnoses are also used to discern the bruxism cohort, in which such a diagnosis led to exclusion. As an additional tool to assess trial eligibility, the GCPS is applied in the TMD cohort as a short, reliable, and valid instrument [38] for which a correlation with splint treatment effects was established [39]. Patients with GCPS grade III and IV are excluded since these scores point to high pain and moderate to severe disability that in most cases cannot be sufficiently alleviated by splint treatment and instead require multimodal pain therapy [39].

The bruxism and TMD cohort are then investigated for differences in OHRQoL and participant satisfaction with the splints in relation to their fabrication mode. OHRQoL was chosen as the primary outcome parameter, as OHRQoL with the splints determines participant compliance and thus the use of the splint, which consequently has an impact on the therapeutic efficacy and the technical outcome of the splint. The assessment of OHRQoL, therefore, forms the base for the future selection of treatment options enabling a maximum of treatment comfort and benefit for the patients [40]. For assessment of OHRQoL, the OHIP-G14 is used, which is an established questionnaire focusing on the evaluation of oral function, orofacial pain, orofacial appearance, and psychological impact [41–44]. To further integrate questions that specifically focus on participant satisfaction with the splints, participants are asked to rate wear comfort and retention of the splints on a Visual Analog Scale (VAS). Therapeutic efficacy and technical outcome are evaluated since these parameters determine clinical applicability as well as the service life of the splints and are therefore—in conjunction with OHRQoL—important measurements for the cost and benefit ratio. Especially for the GCPS, it was shown that it allows monitoring of even small changes in TMD symptoms [20] turning it into a sensitive instrument to validate therapeutic efficacy.

This trial will thus provide important information on key aspects of the clinical outcome of 3D printed stabilization splints that will benefit future individuals with bruxism and TMD and help dentists decide which fabrication method is preferable for a particular patient. However, since 3D printed splint materials show different mechanical properties like hardness, flexibility, and fracture toughness [45], the results of the present study may not be applicable to all types of 3D printed splints. The authors, therefore, hope to encourage further research in this field investigating the clinical outcomes of different 3D printed stabilization splints.

## Trial status

The protocol version 1.1 from the 22^nd^ of December 2023 was approved by the Ethics Committee of the Albert-Ludwigs-University, Freiburg, Germany, on the 18^th^ of January 2024. Recruitment began thereafter and is planned to be completed in September 2024.

## Data Availability

The data generated during and/or analyzed during the trial will be available from the corresponding author on reasonable request.

## Abbreviations

TMD: Temporomandibular disorder
TMJ: Temporomandibular joint
PMMA: Polymethylmethacrylate
CAM: Computer-aided manufacturing
BSI: Bruxism screening index of the of the German Society of Craniomandibular Function and Disorders
DGFDT: German Society of Craniomandibular Function and Disorders
DC/TMD: Diagnostic Criteria for Temporomandibular Disorders
OHIP-G14: Oral Health Impact Profile with 14 items
GCPS: Graded Chronic Pain Scale version 2.0
PI: Principal investigator
SPIRIT: Standard Protocol Items: Recommendations for Interventional Trials (SPIRIT).
INfORM: International Network for Orofacial Pain and Related Disorder Methodology
MDR: Medical Device Regulation
